# Development of Ni–Mo carbide catalyst for production of syngas and CNTs by dry reforming of biogas

**DOI:** 10.1038/s41598-023-38436-8

**Published:** 2023-08-09

**Authors:** Supanida Saconsint, Atthapon Srifa, Wanida Koo-Amornpattana, Suttichai Assabumrungrat, Noriaki Sano, Choji Fukuhara, Sakhon Ratchahat

**Affiliations:** 1 Department of Chemical Engineering, Faculty of Engineering, Mahidol University, Nakhon Pathom, 73170 Thailand; 2 Department of Chemical Engineering, Faculty of Engineering, Center of Excellence in Catalysis and Catalytic Reaction Engineering, Chulalongkorn University, Bangkok, 10330 Thailand; 3 Department of Chemical Engineering, Faculty of Engineering, Kyoto University, Kyoto, 615-8510 Japan; 4 Department of Applied Chemistry and Biochemical Engineering, Graduate School of Engineering, Shizuoka University, Shizuoka, 432-8561 Japan

**Keywords:** Environmental sciences, Energy science and technology, Engineering, Materials science, Nanoscience and technology

## Abstract

Biogas has been widely regarded as a promising source of renewable energy. Recently, the direct conversion of biogas over heterogeneous catalysts for the simultaneous production of syngas and carbon nanotubes exhibits a high potential for full utilization of biogas with great benefits. Involving the combined dry reforming of methane and catalytic decomposition of methane, the efficiency of process is strongly depended on the catalyst activity/stability, mainly caused by carbon deposition. In this study, Ni–Mo catalyst is engineered to provide a life-long performance and perform high activity in the combined process. The surface modification of catalysts by a controlled carburization pretreatment is proposed for the first time to produce a carbide catalyst along with improving the catalyst stability as well as the reactivity for direct conversion of biogas. The performance of as-prepared carbide catalysts is investigated with comparison to the oxide and metallic ones. As a result, the Ni–Mo_2_C catalyst exhibited superior activity and stability over its counterparts, even though the condensed nanocarbon was largely grown and covered on the surface. In addition, up to 82% of CH_4_ conversion and 93% of CO_2_ conversion could remain almost constant at 800 °C throughout the entire test period of 3 h under a high flowrate inlet stream of pure biogas at 48,000 cm^3^ g^−1^ h^−1^. The XPS spectra of catalysts confirmed that the presence of Mo_2_C species on the catalyst surface could promote the stability and reactivity of the catalyst, resulting in higher productivity of carbon nanotubes over a longer time.

## Introduction

The drastic increase in the world’s population and the radical industrial transformation are considered the major reason for exponential growth in total energy demand. Indeed, a gigantic portion of global energy supplies is dominated by fossil fuel combustion, which is expected to be one of the crucial sources of CO_2_ emissions and leads to environmental issues. In the meantime, hydrogen is getting further recognition around the globe as an energy carrier and as a potential fuel that promises to reduce the consumption of these conventional fossil fuels. Moreover, it offers pollution-free solutions for the sustainable development of fuel cell technology^1–3^. At present, there are numerous feasible pathways that have been proposed for hydrogen production such as steam reforming, partial oxidation, coal gasification, hydrocarbon pyrolysis, and so on^4–7^. Apart from the traditional renewable energy productions, dry reforming of methane (DRM) in Eq. (1) is being increasingly studied and stands as the most attractive way for utilizing biogas and producing syngas mixtures (H_2_ + CO). This is because it can provide syngas with H_2_/CO ratio close to 1.0, particularly on account of the abatement of two of the primary substantial greenhouse gases^8,9^.

1 $${\text{DRM}}:\;{\text{CH}}_{{4}} + {\text{CO}}_{{2}} \to {\text{2CO}} + {\text{2H}}_{{2}} \quad \Delta {\text{H}}_{{{298}\;{\text{K}}}} = + {247}{\text{.3}}\;{\text{kJ/mol}}$$

Active metals from groups like Ru, Rh, Ir, Pd, and Pt, have been found to perform superior reactivity and stable performances with high resistance to coke formation in DRM. On the one hand, the uneconomical cost of these precious metal catalysts and their restricted availability make them unsuitable for commercial upscaling applications^10^. Supported Ni-based catalysts, on the other, are much preferably suggested due to their affordable price and comparable activity to those of noble metals^11–14^. Nevertheless, the deactivating of the active phase affected by carbon poisoning taking place mainly in the reforming process is the primary intensive drawback for such reforming catalysts^15^. In this regard, most studies have been conducted with a focus on the way to prevent these unwanted carbonaceous deposits on the catalyst surface^16–19^. In our previous work^20^, we successfully proposed an effective approach to overcome this momentous concern by transforming the deposited carbon into valuable nanomaterials such as carbon nanotubes (CNTs), which can be simultaneously promoted alongside the catalytic decomposition of methane (CDM) reaction, as shown in Eq. (2). The results demonstrated that the use of bimetallic Ni–Mo/MgO catalysts showed catalytically outstanding performance at 800 °C in the conversion of biogas into H_2_-rich syngas and multi-walled carbon nanotubes (MWCNTs) via the integrative processes between dry reforming of methane and catalytic decomposition of methane. Even so, in the long-term practical point of view, a great drop in CH_4_ and CO_2_ reaction rates seemed to become clear. This degradation behavior can be explained by catalyst deactivation while performing in a 20 h run. Hence, there is a desire to further develop a catalyst that is low-cost, well-qualified, and allowed to withstand for an extended duration.

2 $${\text{CDM}}:\;{\text{CH}}_{{4 }} \to {\text{C}}_{{\text{(s)}}} + {\text{H}}_{{2}} \quad \Delta {\text{H}}_{{\text{298K }}} = + {74}{\text{.9}}\;{\text{kJ/mol}}$$

A short while ago, the arisen temptation in the interstitial compounds displayed in transition metal carbides (TMCs) has promisingly driven research as an alternative material for this purpose because they can act similarly or even greatly better than that of platinum in a variety of reactions including the reforming of hydrocarbon species, hydrogenation, desulfurization, isomerization, and biomass conversion^21–25^. These early classes of catalysts can also possess non or less amount of coking compared with traditional reduced Ni catalysts, which suffered from these serious problems^26^. As pointed out in the aforementioned existing literature reviews, the carbides of molybdenum and tungsten (Mo_2_C and WC) are the popular choices among all the available TMC materials applied for DRM reaction. Claridge et al.^27^ illustrated that the highly stable activity of more than 72 h operation could be achieved over β-Mo_2_C at elevated pressures (8 bar). However, carbides were found to deactivate immediately corresponding to the transformation of Mo_2_C to an inactive MoO_2_ phase via its bulk oxidation with CO_2_ (Eq. 3) at atmospheric pressure, as observed by York et al.^28^. The physicochemical properties of carbide and oxide phases may change alternatively based on whether it is in the presence of CO_2_ or CH_4_. This redox mechanism is so-called the “oxidation-carburization” cycle^29,30^. As a result, it is worth noting that at ambient pressure the reaction of CO_2_ with monometallic carbides was considerably preferred and resulted in losing their activity by the oxidation of carbide catalysts. Such kind of issue can be solved by introducing nickel (Ni) to carbides because it is widespread to facilitate the dissociation of CH_4_ (Eq. 4) in the carbide-cycled system. Thus, it is possible to promote the regenerated form of Mo_2_C, which is responsible for maintaining the catalyst stability^29,31–36^.

3 $${\text{Oxidation}}:\;{\text{Mo}}_{{2}} {\text{C}} + {\text{5CO}}_{{2}} \to {\text{2MoO}}_{{2 }} + {\text{6CO}}$$

4 $${\text{Carburization}}:\;{\text{2MoO}}_{{2}} + {\text{5CH}}_{{4}} \to {\text{Mo}}_{{2}} {\text{C}} + {\text{4CO}} + {\text{10H}}_{{2}}$$

In this study, Ni–Mo/MgO catalyst is engineered to provide a lifelong performance in dry reforming of methane and catalytic decomposition of methane reactions, as well as yielding syngas with high purity and well-structured carbon nanotubes by emphasizing studying on catalyst pretreatment process. The impact of surface modification of catalysts controlled under different gas environments of O_2_, H_2_, and CH_4_ (denoted as oxide, metallic, and carbide) was examined, and the long-term stability of the as-prepared catalysts was tested. The catalysts acquired before and after the reaction with all the gases and solid products were analyzed thoroughly to better understand the relationship between the characteristics of catalysts and their catalytic performance.

## Experimental

### Synthesis of catalyst

The Ni–Mo catalyst supported on magnesium oxide (MgO) was synthesized by impregnating 7 g of dried MgO nano-powder (Merck, 98%) with a proper matching amount of Ni(NO_3_)_2_·6H_2_O and (NH_4_)_6_Mo_7_·4H_2_O blended aqueous solution (Sigma-Aldrich, 99.99% trace metals basis) using wetness impregnation method. This procedure gives the nominal Ni and Mo metal loadings of 30wt% (Ni/Mo ratio = 1/1 w/w)^20^. Then, the impregnated solvent was evaporated by a hotplate at 80 °C, attempting to remove excess de-ionized water (DI) from the sample. Lastly, the obtained material was calcined in the exposure of air in a muffle furnace at 500 °C for 3 h with a ramp rate of 10 °C/min.

### Pretreatment of catalyst

**Figure 1:**
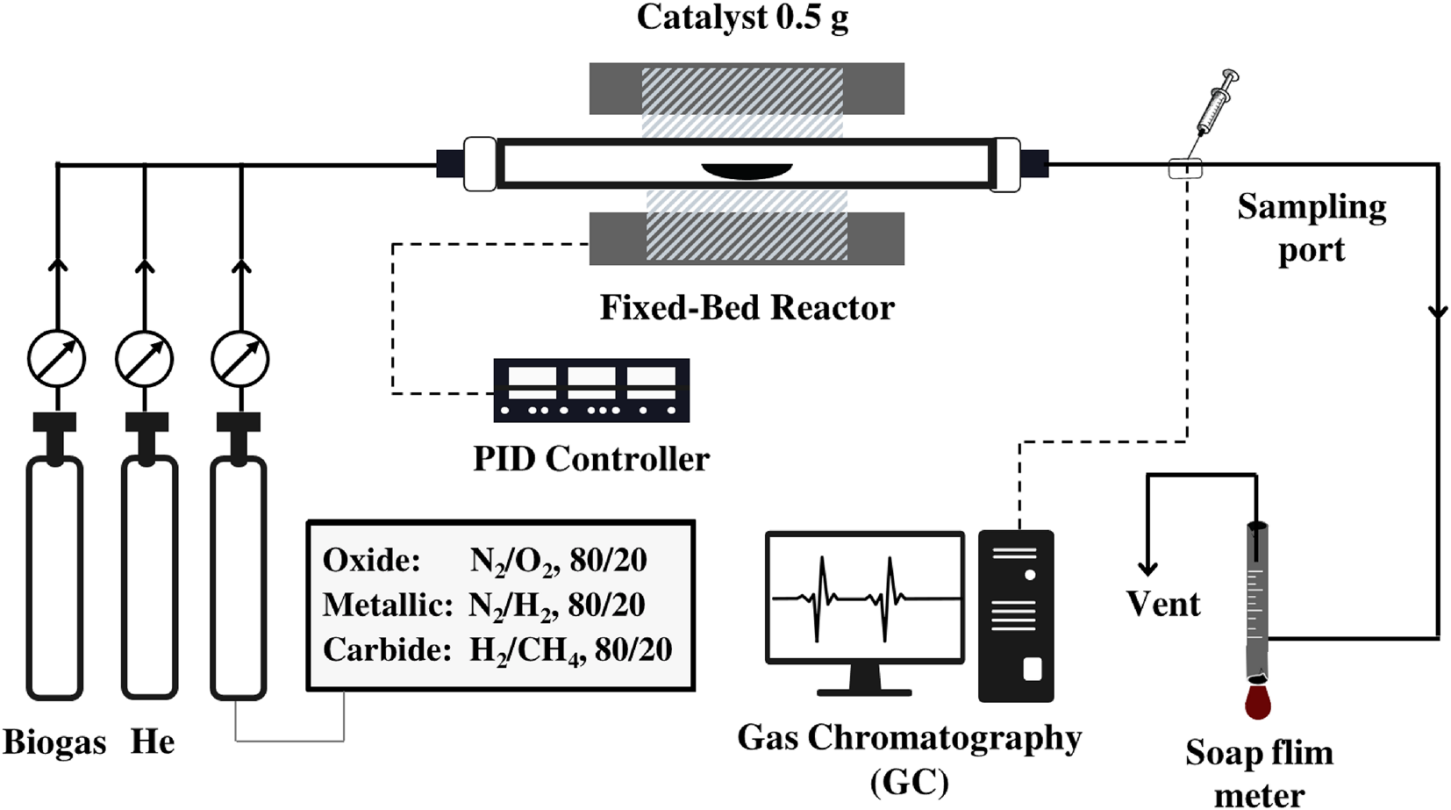
The experimental setup for the production of syngas and carbon nanotubes (CNTs) from biogas.

All the catalytic tests were carried out in a customary fixed-bed reactor at ambient pressure. In the beginning, 0.5 g of as-prepared catalysts filled on a 100 mm long quartz boat were slid into the center of a horizontal 26 mm i.d. tubular quartz reactor (see Fig. 1). A detailed description of the reactor has been previously stated in several works in the literatures^20,37^. For the subsequent proceeding, the oxide precursor (NiMoO_x_) was treated under a variant of activating agents (O_2_, H_2_, and CH_4_) at a feed flow rate of 100 ml/min to achieve the target catalysts. In the meantime, the system was heated from room temperature to 700 °C at a rate of 5 °C/min and then kept at this temperature for 1 h. The resultant Ni–Mo/MgO bimetallic catalysts with a series of various active phases (oxide, metallic, and carbide) were understandably marked hereinafter as NM-O, NM-R, and NM-C, respectively.

### Production process of syngas and CNTs

Prior to the evaluation, the catalyst was pretreated and subsequently heated to 800 °C in a flowing of 60% CH_4_ and 40% CO_2_, which was allowed to continuously pass through along the reactor at a feed rate of 400 ml/min (GHSV = 48,000 cm^3^ g^−1^ h^−1^). The outlet gas steam was sampled regularly at 10 min intervals and analyzed by gas chromatography (TCD, Shimadzu, GC-2014). Finally, after completing a run, the reactor was cooled down in the atmosphere with an inert He flow of 50 ml/min, and in cases where solid carbon produced on the catalyst surface was later collected and preserved for characterization. The reaction time for the activity test was set for 3 h, while the stability was extended to 24 h.

### Evaluation of catalyst activity and stability

The catalytic evaluation of catalysts for the combined reaction of dry reforming of methane and catalytic decomposition of methane, along with the quantitative numerical product analysis was calculated according to the following equation:

5 $$ {\text{CO}}_{{2}} \;{\text{conversion }}\left( {\text{\% }} \right){ = }\frac{{\left[ {{\text{CO}}_{{{2,}\;{\text{inlet}}}} } \right] \times {\text{F}}_{{{\text{inlet}}}} - \left[ {{\text{CO}}_{{{2,}\;{\text{outlet}}}} } \right] \times {\text{F}}_{{{\text{outlet}}}} }}{{\left[ {{\text{CO}}_{{{2,}\;{\text{inlet}}}} } \right] \times {\text{F}}_{{{\text{inlet}}}} }} \times {100} $$

6 $$ {\text{CH}}_{{4}} \;{\text{conversion }}\left( {\text{\% }} \right) = \frac{{\left[ {{\text{CH}}_{{{4,}\;{\text{inlet}}}} } \right] \times {\text{F}}_{{\text{inlet }}} - \left[ {{\text{CH}}_{{{4,}\;{\text{outlet}}}} } \right] \times {\text{F}}_{{{\text{outlet}}}} }}{{\left[ {{\text{CH}}_{{{4,}\;{\text{inlet}}}} } \right] \times {\text{F}}_{{{\text{inlet}}}} }} \times {100} $$

7 $${\text{H}}_{2} \;{\text{yield}}\;\left( {{\text{vol}}\% } \right) = \frac{{{ }\left[ {{\text{H}}_{{{2,}\;{\text{outlet}}}} } \right] \times {\text{F}}_{{{\text{outlet}}}} }}{{{2} \times \left[ {{\text{CH}}_{{{4,}\;{\text{inlet}}}} } \right] \times {\text{F}}_{{{\text{inlet}}}} }} \times {100}$$

8 $$ {\text{Syngas}}\;{\text{purity}}\;\left( {\text{\% }} \right){ = }\frac{{{\text{H}}_{{{2,}\;{\text{outlet}}}} + {\text{CO}}_{{{\text{outlet}}}} }}{{{\text{CH}}_{{{4,}\;{\text{outlet}}}} + {\text{CO}}_{{{2,}\;{\text{outlet}}}} + {\text{H}}_{{{2,}\;{\text{outlet}}}} + {\text{CO}}_{{{\text{outlet}}}} }} \times {100} $$

9 $${\text{CNTs}}\;{\text{yield }}\left( {\frac{{{\text{gCNTs}}}}{{{\text{gCat}}{.}}}} \right) = \frac{{{\text{m}}_{{{\text{product}}}} - {\text{ m}}_{{{\text{catalyst}}}} }}{{{\text{m}}_{{{\text{catalyst}}}} }}$$

10 $$ {\text{CNTs}}\;{\text{percent}}\;{\text{yield}}\;\left( {\text{\% }} \right) = \frac{{{\text{m}}_{{{\text{product}}}} - {\text{ m}}_{{{\text{catalyst}}}} }}{{{\text{m}}_{{{\text{carbon,}}\;{\text{feed}}}} }} \times {100} $$

11 $$ {\text{CNTs}}\;{\text{purity }}\left( {\text{\% }} \right){ = }\frac{{{\text{m}}_{{{\text{product}}}} - {\text{m}}_{{{\text{catalyst}}}} }}{{{\text{m}}_{{{\text{product}}}} }} \times {100} $$

where m_product_, m_catalyst_, and m_carbon, feed_ represent a mass of solid product, initial weight of the fresh catalyst, and total carbon in the feed stream (based on methane), respectively.

### Characterization

The N_2_ adsorption–desorption isotherms were used to measure the specific surface area and pore size distribution of catalysts, using the BET and BJH methods in a Micromeritics TriStar II 3020 analyzer. The chemical composition in the prepared catalysts was confirmed by inductively coupled plasma optical emission spectroscopy (ICP-OES, PerkinElmer) equipment using a Avio™ 550 Max spectrometer. Prior to that, each material was completely mineralized using mixed acids of HNO_3_ and HCl (1:1 v/v). The surface chemistry of the catalyst samples was studied via Mo 3d spectrum measured by X-ray photoelectron spectroscopy (XPS) investigations using a Kratos Axis ultra (DLD) equipped with an Al Kα X-ray source. To determine the crystalline phases within the catalyst structure, the X-ray diffraction (XRD) analysis was carried out on a Bruker D2 Phaser X-ray powder diffractometer, from 10° to 80° (2θ). Moreover, the SEM and TEM micrographs recorded in the field-emission scanning (FE-SEM, JEOL, JSM-7610F) and high-resolution transmission electron microscope (HR-TEM, JEOL, JEM-2100 Plus, Japan) analyses allow the identification of morphology and internal structure on the surface of materials. In the case of carbon products generated during the reaction, the purity and quality of CNTs were further characterized. The purity of synthesized CNTs was calculated from TGA curves. The experiment was done with a Mettler Toledo TGA/DSC 3 + LF instrument. Approximately 3 mg of each sample was heated from room temperature to 900 °C with a heating rate of 10 °C/min under O_2_ flow of 50 ml/min. Lastly, Raman spectrophotometer (PerkinElmer® Spectrum™ GX) was used to quantify the crystallinity and graphitization degree of structure of nanomaterials.

## Results and discussion

### Physicochemical properties of the catalysts

**Figure 2:**
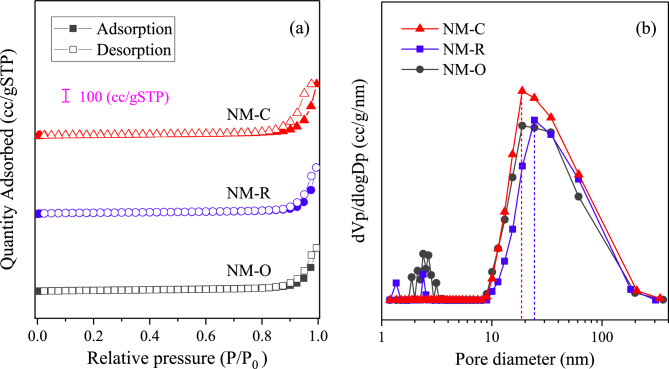
(**a**) Nitrogen adsorption–desorption isotherms and (**b**) BJH pore size distribution of the catalysts after pretreatments.

The textural properties and chemical compositions of the catalysts were characterized by BET and ICP analyses. The N_2_ adsorption–desorption isotherms of the catalysts under pretreated conditions are reported in Fig. 2a. It is shown that the isotherms of all samples are presented in type IV with a large portion of N_2_ gas adsorbed at high relative pressure (P/P_0_), ascribed to the existence of mesopores accompanied by macroporous structure. These hysteresis characteristics are also classified as a H3-type hysteresis loop, which is often ascribed to porous materials made up of slit-shaped with non-rigid aggregates of plate-like pores, according to IUPAC classification^38^. It is suggested that materials that give rise to pore diameters less than 2.0 nm are referred to the micro-structured pores, while the occurrence of pores having a size in the range of 2.0–50.0 nm is associated with mesopore structure. Figure 2b displays the pore size distribution of various catalyst samples obtained by the BJH method. This is confirmed by the curves that all catalysts show two main distribution regions with the predominant and highly strong peak at the average pore diameter ranging between 18.9 and 34.4 nm, which is implied that the majority of porosity in the catalysts is in the characteristic range of mesoporous materials. Textural properties of each catalyst, i.e., the specific surface area (S_BET_), the volume of mesopores (V_meso_), total pore volume (V_total_), and mean pore diameter (D_pore_) are given in Table 1. In comparison with a catalyst in the reduced form (NM-R), the oxide of Ni–Mo/MgO catalyst (NM-O) provides a higher BET specific surface area of 54.6 m^2^/g. When those oxides were reduced into metallic Ni phases with H_2_ flow, their surface area and pore volume declined to 47.9 m^2^/g and 0.537 cm^3^/g. This indicated that there was a blockage of some pores in the NM-R sample. On the contrary, a significant improvement in BET surface area and pore volume was observed for the NM-C catalyst (49.8 m^2^/g and 0.644 cm^3^/g), which consequently may give a positive effect on the catalyst performance by improving the catalytic activity of the catalysts^39,40^. Corresponding to the ICP results, it is certified that the actual weight percentages of Ni and Mo metal are almost equivalent to the nominal value ones.

**Table 1:** Textural properties and elemental compositions of the catalysts after pretreatment.

Catalyst	BET				ICP-OES	
	S_BET_ (m^2^/g)	V_meso_ (cm^3^/g)	V_p_ (cm^3^/g)	D_pore_ (cm^3^/g)	Ni (wt%)	Mo (wt%)
NM-O	54.6	0.568	0.569	18.9	16.3	16.8
NM-R	47.9	0.537	0.538	24.4	15.7	16.2
NM-C	49.8	0.644	0.645	18.9	16.7	17.2

**Figure 3:**
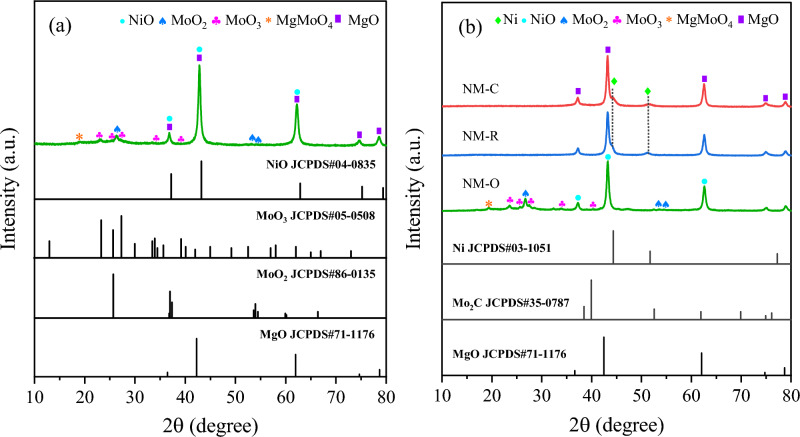
XRD patterns of the catalysts after (**a**) calcination and (**b**) pretreatment stage.

The XRD diffractograms of the catalysts prepared after calcination and pretreatment under varied conditions are shown in Fig. 3. As for all the catalyst samples, it can be found that several diffraction peaks at 2θ = 37.2°, 43.2°, 62.3°, 74.6°, and 78.6°, are predominantly ascribable to (111), (200), (220), (311), and (222) reflection planes of magnesium oxide support (JCPDS 45-0946) with high crystalline, which is proved by a sharp peak of XRD results. In case of treating catalyst in a flow of O_2_, the XRD signal exhibits noticeably similar to that obtained from the fresh calcined catalyst at 500 °C for 3 h. On the other hand, it was noticed that two reflection peaks assigned to metallic phases of Ni (JCPDS 03-1051) were clearly detected at 2θ values of 44.4° and 51.5° as to NM-C and NM-R. No other obvious characteristic peaks of their metal oxides were observed in both cases. These results evidence that the conversion of Ni^2+^ into active Ni^0^ metals could be properly reduced by use of H_2_ and CH_4_ activating gases. The low intensity of the diffraction features might be contributed to a high dispersion ability of Ni in the support^41^. In addition, through the carbothermal treatment of MoO_x_ in a 20 vol% CH_4_/H_2_ gas mixture, the formation of Mo_2_C would appear. However, as elucidated in Fig. 3b, the diffraction peaks of Mo_2_C species cannot be identified in the carbide catalyst, perhaps owing to the small loading of molybdenum and/or a highly dispersed state of carbide phase^42^.

**Figure 4:**
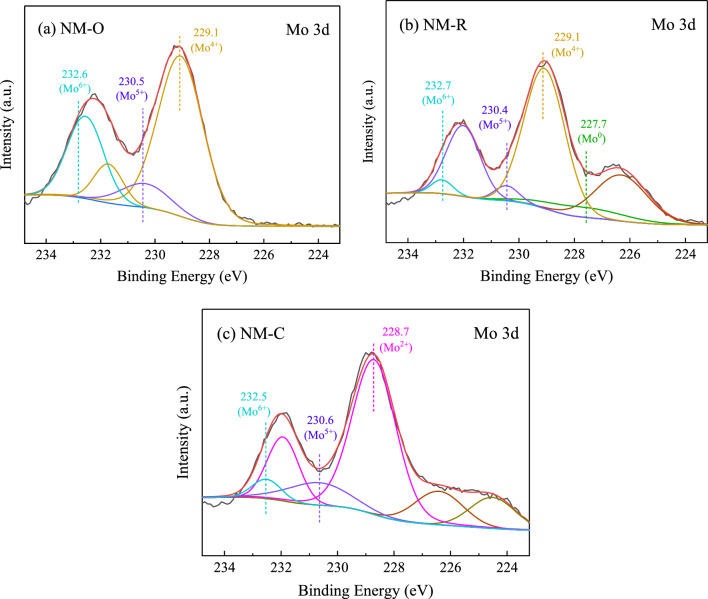
XPS spectra of Mo 3d over catalysts with different catalytic phases.

X-ray photoelectron spectroscopy (XPS) measurement was employed in essence to study the chemical states and elemental compositions within a material or covering its surface. The Mo 3d spectrums of as-prepared catalysts analyzed by XPS are exemplified in Fig. 4. The curve-fitting of Mo 3d profiles was performed to observe the distribution of molybdenum oxidation states and some information on these peaks was estimated and summarized in Table 2. With respect to the observation of collected results in all catalysts, the doublet peaks should split into 3d_5/2_ and 3d_3/2_ of Mo core levels with a spin energy separation of roughly around 3.0–3.2 eV, which corresponds to the coupling effect of spin-orbital^43^. Over the sample of NM-O, it is found that only the characteristic peaks of molybdenum oxide phases appeared at the surface region of Mo^4+^ (229.1 eV), Mo^5+^ (230.5 eV), and Mo^6+^ (232.6 eV) 3d_5/2_ transitions involved in Mo–O bonding^44–46^. The result reveals that the surface oxidation of a catalyst can be completely achieved in a flow of O_2_-containing gas stream. In Fig. 4b, the peaks assigned to MoO_2_, Mo_2_O_5_, and MoO_3_ notably decreased, while fabricating a new peak with Mo 3d_5/2_ binding energy (B.E.) of 227.7 eV, which contributes to the reduced metallic Mo^0^ phase^47^. The main Mo species detected in NM-C is dominated by the molybdenum atoms in the carbidic phase arranged at lower oxidation states ranging from 228.4 to 228.7 eV, involving the Mo–C bond^48^. Considering in Fig. 4c and Table 2, a much smaller concentration of Mo^5+^ and Mo^6+^ at 230.6 and 232.5 eV, as well as the almost absence of Mo^4+^ species were evidenced on the surface of the carbide catalyst. As previously reported elsewhere, the presence of these oxides could be explained by surface oxidation during the passivation process^36,49–51^. However, none of these catalysts was passivated in the exposure to air prior to the XPS analysis. In this circumstance, it is possible to conclude that some of the oxides remaining in the catalyst should be owing to the incomplete carburization^35,52^. As demonstrated in Table 2, the value of molybdenum carbide to total molybdenum species also affirmed that although there were Mo oxides presented during the process, the overall portion of molybdenum species is in the form of carbide.

**Table 2:** Curve fitting results of Mo 3d profiles of as-prepared catalysts.

Catalyst	Peak area					Mo^2+^/ (Mo^0^ + Mo^2+^ + Mo^4+^ + Mo^5+^ + Mo^6+^)
	Mo^0^ (metallic)	Mo^2+^ (carbide)	Mo^4+^ (oxide)	Mo^5+^ (oxide)	Mo^6+^ (oxide)	
NM-O	–	–	4828.4	750.5	1885.2	–
NM-R	426.9	–	2950.4	185.3	149.8	–
NM-C	–	3579.6	–	725.4	273.7	0.78

### The existence of Mo_2_C species

**Figure 5:**
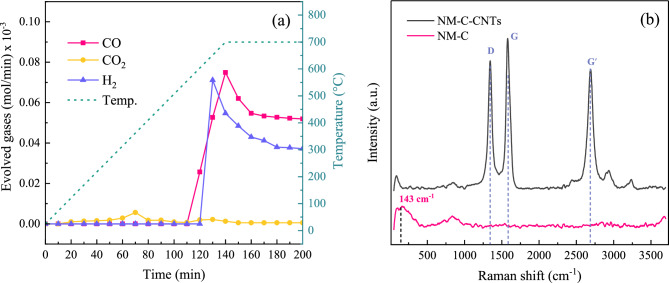
(**a**) the profiles of the gases evolved during the carburization as a function of time. (**b**) Raman spectra of fresh carbide catalyst after exposure to a CH_4_ flow at 700 °C compared with the post-reaction NM-C catalyst.

To further investigate the phenomena involved in the Mo_2_C formation, an in-situ study of gas evolution during the carburization process was conducted. The catalyst was exposed to a CH_4_/H_2_ constant flow of 100 ml/min at an elevated temperature of 700 °C. The outlet concentration of evolved gases was analyzed by GC-TCD detector at intervals of 10 min and the results are illustrated in Fig. 5a. It can be observed that CO and H_2_ were being increasingly produced from the outlet stream after 110 min time (around 600 °C). It is known that carbides of molybdenum (Mo_2_C) commonly form at temperatures above 500 °C^53^. According to Eq. (4) and Fig. 5a, the production of CO and H_2_ at higher temperatures could be attributed to the oxide-carbide transformation. During the carburization, the reduction of nickel oxide can simultaneously occur, (Eq. 12), as evidenced by the presence of Ni in the XRD pattern of fresh NM-C catalyst. In addition, the experimental results surprisingly showed a small amount of CO_2_ generated in the outflow at the initial carburizing time. This may be related to the water–gas shift reaction (WGS), represented in Eq. (13). The reaction consumes all the H_2_O that was produced by the reduction of NiO. Since WGS is an exothermic reaction, thus low temperatures are more favorable^54^, resulting in a slightly detectable CO_2_ at 0–110 min. After the in-situ observations, the NM-C catalyst sample was characterized by using Raman analysis. From the spectra in Fig. 5b, the peak at 143 cm^−1^ also confirms the existence of Mo_2_C species on the surface of the NM-C catalyst and no other characteristic band of pyrolytic carbons was observed.

12 $${\text{Reduction}}:\;{\text{NiO}} + {\text{H}}_{{2}} \to {\text{Ni}} + {\text{H}}_{{2}} {\text{O}}$$

13 $${\text{Water}}\;{\text{Gas}}\;{\text{Shift}}\;\left( {{\text{WGS}}} \right):\;{\text{CO}} + {\text{H}}_{{2}} {\text{O}} \to {\text{CO}}_{{2}} + {\text{H}}_{{2}}$$

### Catalytic performances

**Figure 6:**
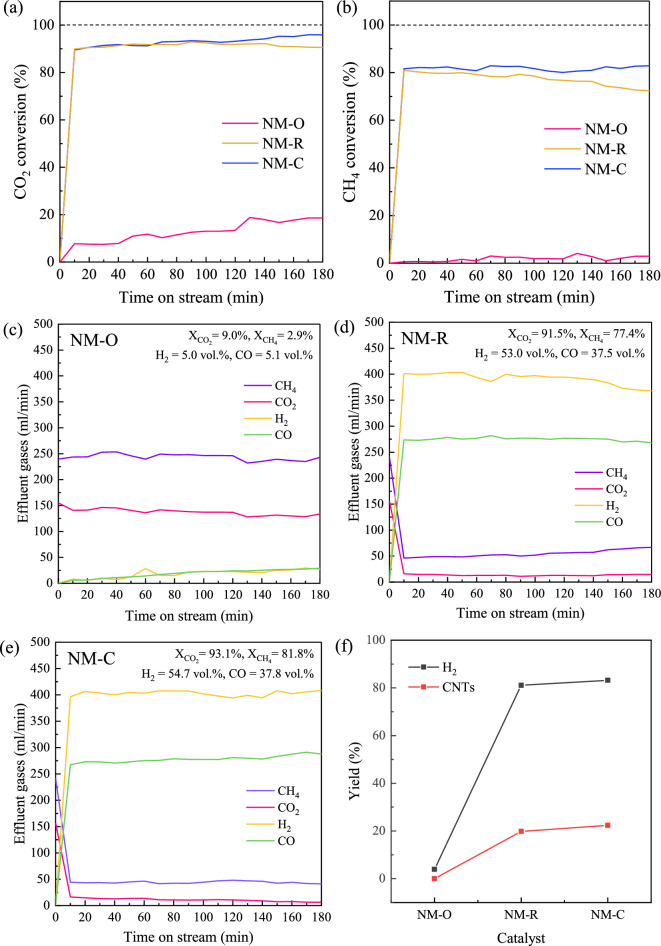
Catalytic performances during time on stream of 3 h at 800 °C, GHSV = 48,000 ml/g-h: (**a**) CO_2_ conversion, (**b**) CH_4_ conversion, (**c**–**e**) effluent gases, and (**f**) H_2_ and CNTs production yields over oxide, metallic, and carbide forms of Ni–Mo/MgO catalysts.

The performance variation of NM-O, NM-R, and NM-C catalysts demonstrated in terms of activity, selectivity, and stability as a function of time on stream (TOS) during a 3 h reaction is shown in Fig. 6. From Fig. 6a, it was obvious that NM-R and NM-C showed a similar trend in the rate of CO_2_, with approximately 90% conversion which remained constant throughout the reaction time. However, a rapid decrease in CH_4_ conversion was observed over the reduced Ni–Mo/MgO (see Fig. 6b). Meanwhile, Ni–Mo_2_C/MgO exhibits stable performance with a slightly higher CH_4_ conversion than that of NM-R over the entire test period. This could be implied that the presence of Mo_2_C species may have a significant improvement in the catalyst activity and durability. In contrast, the CO_2_ was relatively converted through the dry reforming of methane reaction for the oxide form of the catalyst. Also, an almost undetectable conversion of CH_4_ and no carbon deposition indicates that the catalyst was unable to activate the decomposition of methane for producing H_2_ and CNTs. Thus, it implies that the NM-O catalyst would favor DRM more than CDM, complying with DRM theoretically producing an equal rate of H_2_ and CO as in Fig. 6c. Table 3 shows the analysis of total moles of carbon in feed and products (gas and solid) together with carbon balance for the 3 h reaction time. It was found that there was only a slow rate of DRM over NM-O (CO = 0.14 mol), and no CDM occurred. Considering carbon products from DRM and CDM (CO, C), it was found that the rate of DRM over NM-R catalyst was 3 times higher than CDM (DRM: CH_4_ + CO_2_ → 2CO + 2H_2_ ; CO = 2.02 mol, CDM: CH_4_ → C + 2H_2_ ; C = 0.37 mol). Compared to NM-R counterpart, NM-C catalyst enhanced the rate of CDM more than DRM as CH_4_ consumed more than CO_2_. The mole of CH_4_ reduced from 0.40 to 0.32 mol, while CO_2_ was slightly reduced from 0.10 to 0.08. It confirms that the existence of Mo_2_C can improve the catalytic activities of both DRM and CDM, but it pronounces the enhancement of CDM than DRM. The exact product output data of gas and solid phases derived using different catalysts after a constant reaction time of 3 h are summarized in Table 4. Compared to NM-O, it was discovered that the yield of H_2_ greatly increased when the NM-R and NM-C were used, attributed to the presence of Ni^0^ enhancing CH_4_ dissociation. Theoretically, there are two possible pathways for generating hydrogen. One can produce from DRM, while the other is CDM^55^. However, as observed from rates of H_2_ (Fig. 6d,e) thus, it could be said that most of H_2_ produced in this system comes primarily through DRM reaction. As a result, the syngas H_2_/CO ratio of 1.42–1.45 was higher than 1.0. This value is suitable for producing a broad range of clean fuels and valuable hydrocarbon products, including olefins, diesel, gasoline, and so on, via the well-known catalytic chemical process, namely Fischer–Tropsch (FT) synthesis^56^. In the case of solid carbon shown in Fig. 6f, the production yield of carbon nanotubes shows a similar trend as the same as H_2_ yield, relating to CDM reaction. Furthermore, it is apparent that the largest amount of CNT was synthesized over NM-C. This could be explained by the large pore volume of the catalyst (see Table 1). It has demonstrated that the pore volume of the catalyst may have an important effect on the CNT yield. The high capacity of pore volume promotes the reduction of the barriers to diffusion and transport, which enable biogas molecules to distribute deep into the inner surface of the catalyst and result in a higher CNTs yield^57^. Besides, it has been reported that the Mo_2_C phase enhances the diffusivity of carbon atoms into neighboring Ni nanoparticles, avoiding the encapsulation of carbon deposition and leading to CNT growth^42,58^. As described so far, it is reasonable to deduce that the carbide catalyst not only shows comparable reactivity and durability but is also capable of producing CNTs with the highest yield.

**Table 3:** Total moles of carbon in feed, gas and solid products for the 3 h reaction time.

Catalyst	Total mole of carbon (mol × 10^1^)					% Carbon balance
	Feed, _in_	CO, _out_	CH_4_, _out_	CO_2_, _out_	C_(s)_	
NM-O	2.94	0.14	1.79	1.01	− 0.01	99.9
NM-R	2.93	2.02	0.40	0.10	0.37	98.5
NM-C	2.94	2.05	0.32	0.08	0.42	97.5

**Table 4:** Summary of key results of gas and solid products over different catalysts used.

Catalyst	Gas product			Solid product		
	H_2_ yield (vol%)	H_2_/CO (–)	Syngas purity (%)	gCNTs/gCAT (–)	CNTs yield (%)	Purity (%)
NM-O	5.02	0.99	10.14	–	–	–
NM-R	81.07	1.42	90.75	8.78	19.81	89.77
NM-C	83.18	1.45	92.52	9.95	22.39	90.86

### Chemical, structural, and morphological analyses of CNTs

**Figure 7:**
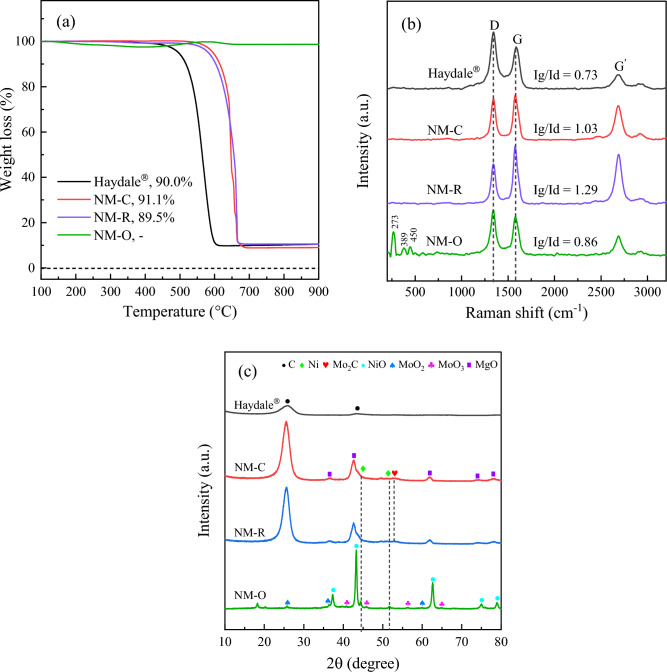
(**a**) TGA profiles, (**b**) Raman spectra, and (**c**) XRD analyses of as-grown CNTs over spent catalysts in comparison with those of the commercial CNTs.

To investigate the quality and purity of CNTs formed over these catalysts, several techniques have been employed. Figure 7a illustrates the TGA data curves for as-obtained CNTs over the spent catalysts. Obviously, a similar oxidation pattern with one dominant slope at the high-temperature region above 600 °C was observed for the NM-R and NM-C samples. It is known that a pronounced peak of weight loss is referred to the amount of deposited carbon burned in oxygen, which can be attributed to the yield of carbonaceous nanomaterials generated on the catalyst surface during the reaction^59^. On top of that, the remaining mass in the TGA of CNTs could be explainable to unreacted catalysts retained in the sample after completing a run of 3 h. It has been reported that such impurities can be removed by various chemical-treated techniques to achieve better-purified CNTs, such as the acid leaching method^60,61^. The CNTs yield calculated from TGA data curves are also attached in the figure. It is observed that NM-C has the highest carbon content compared to the other catalysts. On the contrary, there is no evident weight loss curve due to the oxidation of carbon was presented for the NM-O, showing that CNTs cannot be formed over the NM-O catalyst sample. Additionally, the prominent weight loss curves detected in the TGA profiles of NM-R and NM-C, which reflected at the elevated temperatures show the high thermal stability and graphitization structure of CNTs produced over these catalysts. This is due to the amorphous carbon is typically oxidized at temperatures less than 400 °C^62^.

In Fig. 7b, the Raman spectroscopy measurement has been carried out to further shed light on the crystallinity and quality of the as-produced MWCNTs received over spent NM-O, NM-R, and NM-C catalysts. It is evident that there are three distinct peaks appeared for all samples. The one with the signal detection at the wavelength ranges of 2600–2700 cm^−1^ can be attributed to the G′ peak, which is used to assure the typical characteristics of multiwall carbon nanotubes^63^. The other two absorption bands appearing at around 1345 cm^−1^ and 1586 cm^−1^ were identified as the D peak and G peak, respectively. In general, the D-band is assigned to the structural defects or disordered-structured carbons such as amorphous carbon presented on the extrinsic surface of CNTs walls. Whereas the G-band is related to the tangential stretching vibration of C=C bonded atoms in the aromatic layers of graphene crystalline^64,65^. The relative integral areas of the G and D bands demonstrated in terms of Ig/Id ratio have been widely applied for quantifying the graphitization degree of MWCNTs^20,37,59^. As can be seen from the spectra, the respective Ig/Id ratios of the synthesized CNTs were found to be in the range of 0.86–1.29, while the poorest value of 0.73 was given by the commercial CNTs. The result indicates that all catalysts possess better-graphitized MWCNTs as compared to the commercial one. In addition, three absorption peaks attributed to MoO_3_ were also found at 273, 389, and 450 cm^−1^ for the NM-O catalyst^66^. It is implied that the metal oxide was unable to reduce into a metallic form during the catalytic test, decreasing the catalyst activity. Figure 7c displays the XRD pattern of reacted catalysts. Clearly, the characteristic diffraction peaks of MgO phases were diminished in the patterns of the NM-R and NM-C. As opposed to the XRD profiles of the fresh catalysts, two dominant peaks were observed at 2θ reflections of 26.1° and 42.6° in the spent catalysts, which can be attributed to the characteristic of graphitic carbon and therefore to the formation of carbon nanomaterials (CNTs)^67^. In addition, the peaks attributed to metallic Ni^0^ at 2θ = 43.8° and 51.2° were detected in both samples and there is no peak assigned to their metal oxide was observed. This indicates the complete reduction of Ni oxide into active Ni during the reaction. As for the diffractogram of the NM-O sample, it was found that most of the peaks in the diffraction pattern are in the form of oxide phases, which are in accordance with the previous results provided by Raman spectra.

**Figure 8:**
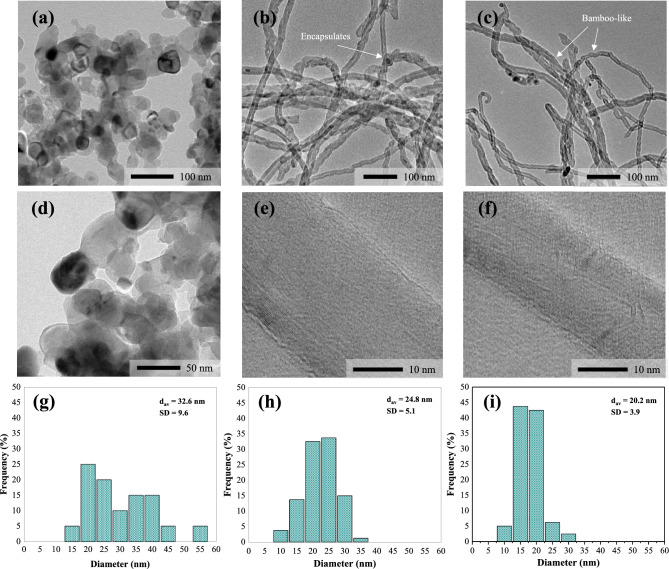
TEM micrographs and PSD histograms of CNTs synthesized over (**a**, **d**, **g**) NM-O, (**b**, **e**, **h**) NM-R, and (**c**, **f**, **i**) NM-C catalysts on MgO support for 3 h at 800 °C, 48,000 ml/g-h.

To further investigate a deep insight into the surface morphological and internal structured differences of carbon deposition after a run of 3 h, the reacted catalysts were characterized by TEM analysis. The recorded micrograph and its magnified version are presented in Fig. 8. The diameter size distribution of nanotubes on the catalysts was estimated based on the TEM images and the results are given in Table 5. As shown in Fig. 8, it was evident that the highly dense CNTs were produced in all catalyst configurations. Only the catalyst sample that was not very active presented a negligible amount of such filamentary nanocarbon. For the NM-R and NM-C, the general nanostructure of deposited carbon is quite similar, consisting of jointed compartments resembling the formation of bamboo plant with hollow stem cavities separated by diaphragms, which represents a morphology typical of filamentous carbon with bamboo-like structure^68–71^. A few segments are assembled with encapsulated catalyst particles, while the others are empty. The number of the graphitic layer in tube walls is varied from 8 to 27 layers and it correlates well with the thickness of the tubes (Table 5). As can be seen, it was shown that the presence of CNTs synthesized over NM-R exhibits mainly those bamboo-like nanostructures with larger particles and irregular morphology. This could be attributed to the sintering effects of catalysts during the reaction, which might be responsible for the deactivation problems. On the opposite hand, small particles with uniform diameters were detected in the micrographs of Ni–Mo_2_C/MgO. The results suggested that the catalytic particles were somehow confined or controlled by carbon layers of carbide species covering the catalyst surface, which was hypothesized to be a possible reason for the reduction in particle agglomeration. Therefore, it is interesting to note that NM-C has a better resistance to particle sintering compared to the conventional reduced form, which can be also responsible for the stabilization of its catalytic performance.

**Table 5:** Summary of morphological parameters analyzed by SEM and TEM images for different catalysts used.

Sample	Type of CNTs	Diameter^a^ (nm)	SD^b^ (nm)	Distribution (nm)	No. of wall
NM-O-CNTs	n.d	–	–	–	–
NM-R-CNTs	MWCNTs	24.8	5.1	13–37	14–27
NM-C-CNTs	MWCNTs	20.2	3.9	12–34	8–18

^a^Average diameter of CNTs.

^b^Standard deviation of nanotube diameter.

### Stability test

**Figure 9:**
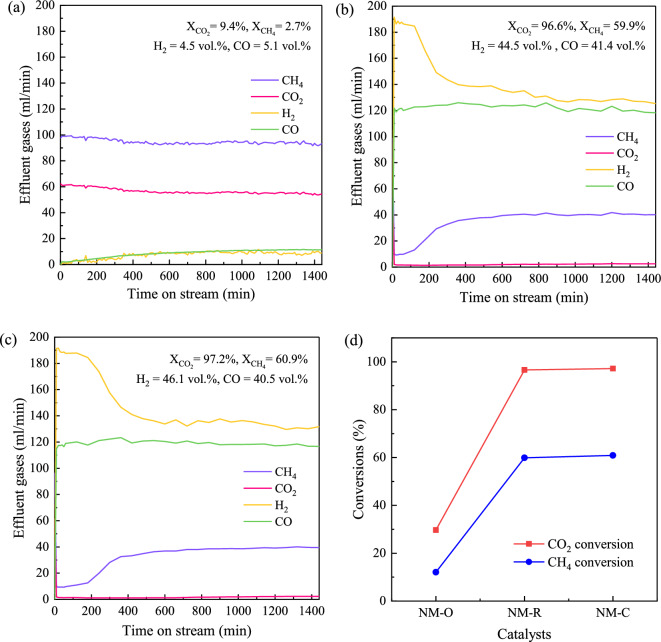
Effluent gases detected throughout the entire reaction over (**a**) NM-O, (**b**) NM-R, and (**c**) NM-C catalysts for 24 h at 800 °C, 48,000 ml/g-h, along with (**d**) CO_2_ conversion and CH_4_ conversion.

The long-term performance of NM-O, NM-R, and NM-C catalysts for the combined process of DRM and CDM was evaluated at a low biogas feeding rate (decreased from 400 ml/min to 160 ml/min) over a 24 h run. The GHSV was fixed at 48,000 ml/g-h (using 0.2 g of catalysts). Figure 9 presents all the possible gases detected in the outlet stream during the stability test at 800 °C. In Fig. 9a, it is obvious that the rate of biogas consumption has not changed much compared to the inlet gas compositions. Meanwhile, the rate of H_2_ generation slowly increased and after 800 min of TOS, the effluent gases of H_2_ show a constant trend at a low concentration of around 10–11%. A possible reason for this phenomenon might be that NM-O could not act promptly as the catalytic role at an early stage of reaction. This is because the active state of these catalysts should be in a metallic form (Ni^0^), which can be achieved under H_2_ flow. When there was enough hydrogen generated out from the stream to complete the reduction of catalysts, the conversion would be improved. For the NM-R and NM-C catalysts, the compositions of effluent gases exhibited similar trends, with approximately 95% of the CO_2_ being completely converted. In addition, a high fraction of CO concentration was produced from the outlet stream and progressively retain constant over 24 h of TOS in both cases, whereas a higher concentration of H_2_ was observed for the NM-C catalyst. Considerably, the carbide catalyst can promote an improved selectivity toward high-purity hydrogen production. It is also demonstrated that the NM-C shows a prolonged catalytic stability, while NM-R could be active in a short period for only 1 h. Then, the production rate of H_2_ decreased rapidly. The main mechanism is due to the presence of individual phases of Ni and Mo_2_C. It is worth noting that the contact between Ni and Mo_2_C phases could exhibit dual-active sites, creating the redox cycle of the carbide phase^35,36^. According to this cycle, CO_2_ in biogas is activated by Mo_2_C, then, the formation of adsorbed O_2_ replaces the carbon of Mo_2_C to MoO_2_. On the other hand, Ni is responsible for the dissociation of CH_4,_ and carbon species formed on Ni sites allow MoO_2_ to be reduced back into Mo_2_C, thus, slow down the easy deactivation of active phases from the sintering as well as coke formation caused by catalytic decomposition of methane, which is responsible for maintaining the catalytic activity and stability of the system during the reaction.

The productivity and properties of the as-grown CNTs on these catalysts were investigated. After completing the 24 h reaction, a solid post-reaction catalyst was collected and calculated in terms of yield, while the purity of CNTs was estimated by TGA measurement. And the relative intensity ratio of Ig/Id acquired from Raman spectroscopy analysis, was applied to evaluate the crystallinity and the graphitization degree of carbon deposition. Table 6 summarizes the yields, purity, and Ig/Id value of synthesized CNTs as well as those corresponding commercial CNTs for comparison. Evidently, there is no carbon nanofilaments are detected on the NM-O catalyst, suggesting that the oxide catalyst could not be functionalized sufficiently to catalyze the CDM under the employed reaction conditions. On the other hand, the highest CNTs yields was achieved over the NM-C catalyst within a 24 h run. So, it is interesting pointing out that the Ni–Mo_2_C catalyst exhibited superior performance over its counterparts, even though the condensed nanocarbon was largely generated and covered on the catalyst surface.

**Table 6:** The yield, purity, and the respective Ig/Id value of as-synthesized CNTs after long-term stability test (24 h, 800 °C).

Catalyst	gCNTs/gCAT (–)	CNTs Yield^a^ (%)	Purity of CNTs^b^ (%)	Ig/Id^c^ (–)
Haydale®	–	–	90.00	0.73
NM-O	n.d	n.d	n.d	0.70
NM-R	16.53	28.45	94.04	1.24
NM-C	20.15	35.52	95.71	0.98

^a^Calculation by weight of solid products with respect to carbon in methane.

^b^TGA analyses.

^c^Intensity ratio of G and D peaks in Raman spectra.

**Figure 10:**
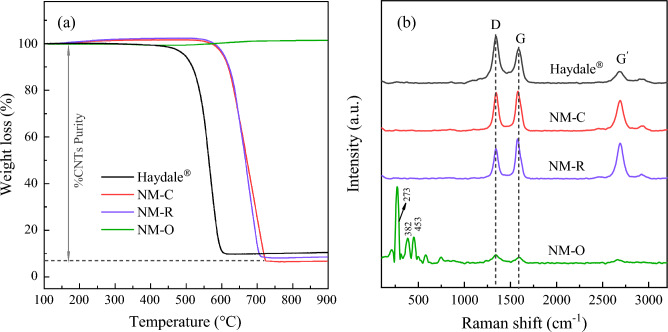
The analyzed data of CNTs produced over a run of 24 h at 800 °C derived by (**a**) TGA and (**b**) Raman analysis in comparison with commercial CNTs.

The TGA profiles, Raman spectra, and X-ray diffraction pattern of CNTs synthesized over spent catalysts after a reaction of 24 h are provided in Fig. 10. As determined from the TGA plots comparing 3 h results, a gigantic portion corresponding to the oxidation with oxygen appeared in the weight loss behavior of NM-C and NM-R samples, suggesting that there was a large number of carbonaceous materials deposited on the catalyst surface, which hence verifies the high purity of as-produced CNTs. This is confirmed by the obtained purity of nearly close to 95.0% over these catalysts, which is also much higher than the features of the 3 h reaction test (around up to 85.0%) and is in line with the results evident in many reports, quoting that the purity of CNTs can be improved by conducting a longer reaction time^20,37,59^. Moreover, the slope of mass disappearance reflected at temperatures of over 550 °C certifies that the CNTs showed excellent properties in terms of thermal stabilization and degree of graphitization structure. In particular, when compared with the commercial CNTs, being thermally stable below 450 °C with lower CNTs purity. On the other hand, the percentage of weight loss due to the burning of CNTs does not change for the thermogravimetric curve of the NM-O catalyst, indicating the absence of such carbon species in the sample. This is in good agreement with the arising of MoO_3_ phases at the Raman shift of 273, 382, and 453 cm^−1^ as shown in Fig. 10b, which present prominently and dominantly amongst the remaining peaks that would assign to the characteristic bands of CNTs. Meanwhile, the data illustrates that the carbon deposited on the NM-C and NM-R catalysts have a more crystalline and well-graphitized structure of CNTs than that on the typical ones since the relative ratios are 0.98 and 1.24, respectively.

**Figure 11:**
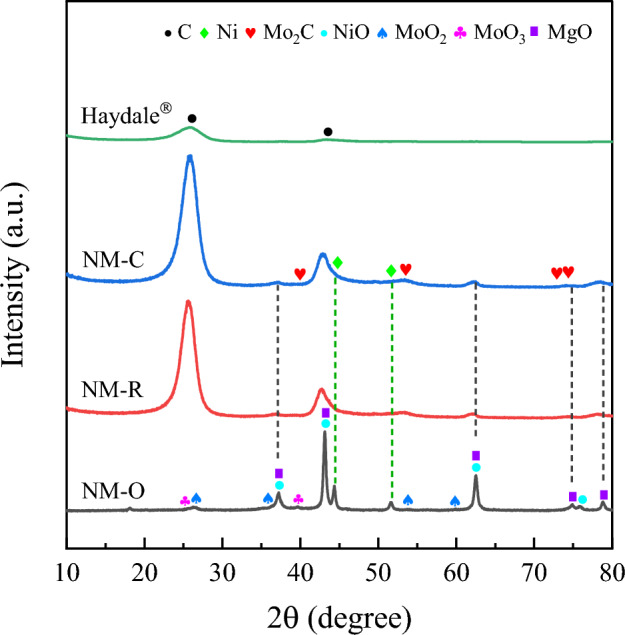
X-ray diffraction patterns of carbon products from a run of 24 h at 800 °C compared with that of commercial CNTs.

**Figure 12:**
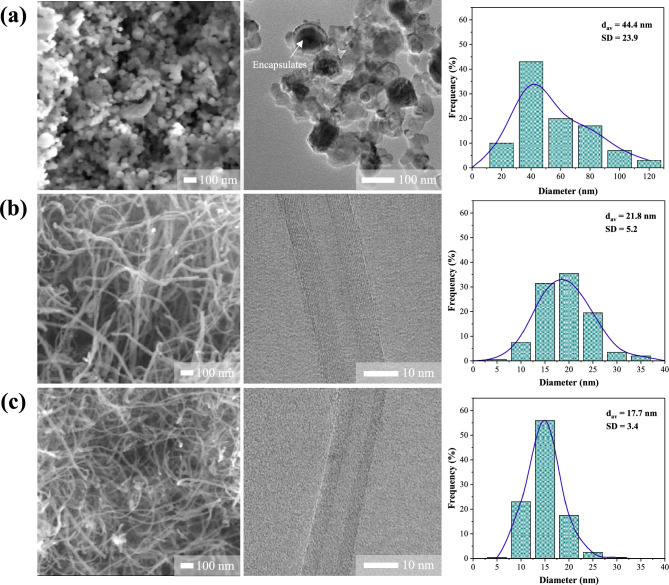
SEM and TEM images with PSD histograms of CNTs produced after a run of 24 h over (**a**) NM-O, (**b**) NM-R, and (**c**) NM-C catalysts.

In Fig. 11, a similar XRD pattern was noticed over the catalysts prepared under a flow of H_2_ and CH_4_ with the main reflection peaks at 2θ = 25.9° and 42.9°, which relates to the appearance of as-synthesized CNTs. On the contrary, the overall proportion of phases derived over the spent NM-O catalyst is in the form of metal oxides and there is no peak attributed to carbonaceous species was observed as virtually elucidated in the SEM and TEM images (see Fig. 12a). This is also in line with the results acquired from TGA and Raman shift. In addition, it should be noted that the molybdenum oxide phase (MoO_2_) is arisen as a consequence of the reduction of Mo^6+^ into Mo^4+^ by hydrogen produced during the reaction. More interestingly, according to the data in Table 7, it was found that the crystallite size of Ni particle of the used NM-C catalyst significantly decreased in comparison with the fresh carbide sample, having 4.7 nm, whereas an increase in particle size from 5.5 to 11.2 nm was observed over NM-R sample. This proves that the carbide catalyst had a greater ability to keep control of the crystal structure. Furthermore, the existence of Ni and Mo_2_C found in the XRD postreaction of the NM-C configuration supports the presumption that there was an oxidation-carburization cycle occurred during the reaction, which is the reason why the system can be maintained for a long time.

**Table 7:** The crystallite size of Ni particles and other components for catalysts derived before and after 24 h reaction determined by XRD.

Sample	Crystallite size (nm)					
	Ni (111)		Mo_2_C (101)	NiO (200)	MoO_2_ (110)	MoO_3_ (040)
	Fresh	Spent				
NM-O-CNTs	n.d	28.9	35.4	22.9	9.5	16.1
NM-R-CNTs	5.5	11.2	6.8	–	–	–
NM-C-CNTs	4.7	8.1	5.8	–	–	–

Figure 12 illustrates the results of SEM and TEM analyses of the post-reaction catalysts in the long-term (24 h) catalytic stability test. All the analyzed morphological parameters calculated based on SEM and TEM characterization are also listed in Table 8. It is clear that there is no sign of carbonaceous nanofilament appearing on the segment of NM-O. In the magnification images, it is understandably seen that most of the active particles were embedded inside some spherical layer, showing the smaller reacting area available, resulting in losing its activity. Also, the CNTs sample generated over NM-O exhibits broad diameters with a large mean particle size of around 44.4 nm. As for the micrographs of Ni–Mo/MgO and Ni–Mo_2_C/MgO catalysts, it is revealed that the white spots in SEM images were barely found in both samples with some of them nucleated at the end of elongated compartments. The most brightness area should be corresponded to the metal particles of the catalyst. Therefore, it can be said that NM-R and NM-C catalysts provided CNTs products with less impurity, which later reduces the cost of related purification processes. Moreover, the CNTs grown over these samples considerably have lower average diameters when compared to those of the 3 h reaction test. Especially on the carbide catalyst, giving the smallest and narrowest number in terms of tube diameter and CNTs wall, which is in accordance with the crystallite data results of XRD, as clearly shown in Table 7.

**Table 8:** Summary of morphological parameters analyzed by SEM and TEM images for different catalysts used.

Sample	Type of CNTs	Diameter^a^ (nm)	SD^b^ (nm)	Distribution (nm)	No. of wall
NM-O-CNTs	n.d	44.4	23.9	17–115	–
NM-R-CNTs	MWCNTs	21.8	5.2	7–36	13–14
NM-C-CNTs	MWCNTs	17.7	3.4	8–30	8–13

^a^Average diameter of CNTs or catalyst.

^b^Standard deviation of CNT or catalyst diameter.

## Conclusion

The catalytic performance of bimetallic Ni–Mo catalysts supported on MgO prepared under different treatment gases (O_2_, H_2_, and CH_4_) was investigated in the process of direct biogas conversion for the simultaneous production of H_2_-rich syngas and CNTs. The experimental results demonstrated that carbide Ni–Mo catalyst prepared by controlled carburization exhibits high reactivity and excellent stability over the long-term test period (24 h). The highest yield CNTs with purity up to 95% can be achieved. In addition, more than 80% of H_2_ yield and 90% syngas purity with H_2_/CO = 1.45 were obtained. By correlating the XPS and Raman results with in-situ study, it is suggested that the layer of carbide species of Ni–Mo_2_C formed on the catalyst surface was primarily responsible for controlling the crystal structure of Ni–Mo and therefore leading to the improved catalytic performance with resultants of the smaller nanotubes of filamentous carbon. On the other hand, a significant decrease in CH_4_ conversion was observed over the conventional reduced Ni–Mo catalyst. As a result, the as-grown CNTs have a much larger size of nanotubes than other catalyst samples due to the sintering. In summary, the presence of Mo_2_C species on the catalyst surface can promote the stability and reactivity of the catalyst for direct biogas conversion. The new findings in this study would accelerate the research and development of direct conversion process of biogas into a stage of commercialization.

## Data Availability

All data related to the finding of this study are accessible upon request from the corresponding author Sakhon Ratchahat.
